# Prevalence of postamputation pain and its subtypes: a meta-analysis with meta-regression

**DOI:** 10.1097/PR9.0000000000000918

**Published:** 2021-05-04

**Authors:** Paul M. Schwingler, Rajat N. Moman, Christy Hunt, Zachary Ashmore, Sandra P. Ogletree, Mason E. Uvodich, M. Hassan Murad, W. Michael Hooten

**Affiliations:** Departments of a Physical Medicine and Rehabilitation and; b Anesthesiology and Perioperative Medicine, Mayo Clinic, Rochester, MN, USA; c Department of Anesthesiology and Perioperative Medicine, Division of Pain Medicine, Mayo Clinic, Rochester, MN, USA; Departments of d Orthopedic Surgery and; e Internal Medicine, Mayo Clinic, Rochester, MN, USA

**Keywords:** Phantom limb pain, Residual limb pain, Postamputation pain, Chronic pain, Prevalence

## Abstract

**Introduction:**

The inconsistent use of standardized approaches for classifying postamputation pain (PAP) has been a barrier to establishing its prevalence.

**Objectives:**

The primary objective of this systematic review and meta-analysis is to determine the prevalence of nontraumatic lower-extremity PAP using an established taxonomy. The secondary objective is to determine the prevalence of PAP subtypes, including phantom limb pain and residual limb pain (RLP).

**Methods:**

An a priori protocol was registered, and a database search was conducted by a reference librarian. Randomized trials and uncontrolled studies were eligible for inclusion. The risk of bias was assessed using a tool developed for uncontrolled studies. A total of 2679 studies were screened, and 13 studies met inclusion criteria (n = 1063).

**Results:**

The sources of risk of bias included selection bias and, to a lesser extent, whether the outcome was adequately ascertained. Two studies reported the prevalence of PAP and the pooled prevalence was 61% (95% confidence interval [CI], 33%–86%) with high heterogeneity (I^2^ = 93%). Thirteen studies reported the prevalence of phantom limb pain and the pooled prevalence was 53% (95% CI, 40%–66%) with high heterogeneity (I^2^ = 93%). Eight studies reported the prevalence of RLP and the pooled prevalence was 32% (95% CI 24%–41%) with high heterogeneity (I^2^ = 76%). Clinical subtypes of RLP were not reported.

**Conclusions:**

The prevalence of PAP is high in patients with nontraumatic lower-extremity amputations. Ongoing research that uses a taxonomy for PAP is needed to fully delineate the prevalence of PAP subtypes.

## Abstract 2

Supplemental Digital Content is Available in the Text. Postamputation pain is high in patients with nontraumatic lower-extremity amputations, but the pooled prevalence rates were associated with high levels of heterogeneity. Ongoing research using the Durham Pain Investigations Group Postamputation Pain Algorithm taxonomy is needed to fully delineate the prevalence of postamputation pain and its subtypes.

## 1. Introduction

Chronic postamputation pain (PAP) is a debilitating condition that stems from a confluence of neurological and musculoskeletal factors. As a result, the prevalence of PAP has been difficult to establish. A related barrier to establishing the prevalence of PAP has been the inconsistent use of standardized approaches for classifying the various clinical conditions responsible for PAP. The Durham Pain Investigations Group PAP Algorithm (DPIG-PAPA) is a taxonomy for PAP based on pain type.^4^ The first 2 subtypes are phantom limb pain (PLP) and residual limb pain (RLP). The latter category is subdivided into a somatic pain subtype (eg, chronic infection, chronic wound inflammation, and prosthesis maladaptation) and a neuropathic pain subtype. The neuropathic pain category is further subtyped as (1) sympathetically mediated pain, often referred to as complex regional pain syndrome-like pain, (2) painful neuroma, and (3) mosaic postamputation neuralgia.^4^

The number of people living in the United States with limb loss is projected to double by the year 2050.^24^ Acquiring detailed knowledge about the prevalence of PAP and its subtypes would enable clinicians, researchers, and policymakers the ability to allocate health care resources based on projections of anticipated need.^6^ Thus, the primary objective of this systematic review and meta-analysis is to determine the prevalence of nontraumatic lower-extremity PAP using an established taxonomy for PAP. The secondary objective is to determine the prevalence of PAP subtypes including PLP and the various subtypes of RLP.

## 2. Methods

### 2.1. Study protocol

This study was deemed exempt by the Mayo Clinic IRB. The Preferred Reporting Items for Systematic Reviews and Meta-Analyses (PRISMA) guidelines^12^ were followed. An a priori protocol was followed. The trial was registered in the PROSPERO database CRD42020159480.^2^

### 2.2. Search strategy

A comprehensive search of several databases from each database's inception to November 20, 2019, was conducted. The databases included Ovid MEDLINE, MEDLINE Epub Ahead of Print, MEDLINE In-Process and Other Non-Indexed Citations, Daily, Ovid EMBASE, Ovid Cochrane Central Register of Controlled Trials, Ovid Cochrane Database of Systematic Reviews, and Scopus. The search strategy was designed and conducted by an experienced librarian with input from the study's principal investigator. Controlled vocabulary supplemented with keywords was used to search for studies of the prevalence of PAP in patients who have undergone lower-limb plus or minus upper-limb amputation. The actual strategy listing all search terms used and how they are combined is available in Appendix A (available at http://links.lww.com/PR9/A105).

### 2.3. Study selection process

Study inclusion criteria included (1) randomized designed, crossover design, and parallel-designed clinical trials, (2) prospective and retrospective observational cohort studies, (3) cross-sectional studies, (4) studies involving adult patients aged ≥18 years, (5) studies from database inception to November 20, 2019, and (6) studies in the English language. Exclusion criteria included (1) studies of patients with chronic limb pain without amputation, (2) studies involving upper-limb amputation only, (3) studies involving acute postoperative pain only, (4) studies of patients with acute postoperative complications (ie, infection, thrombosis, or wound dehiscence), and (5) studies of patients with traumatic amputations only.

Two independent pairs of reviewers screened all titles and abstracts identified by our search strategy in the first phase. In the second phase, the 2 pairs of independent reviewers screened the full text of all studies identified in the first phase and applied inclusion and exclusion criteria. Any disagreements between reviewers with respect to inclusion of studies were resolved by an additional author (R.N.M.).

### 2.4. Data extraction

Data were extracted by 4 independent reviewers using a templated electronic database. Based on the a priori protocol, abstracted data included the prevalence of (1) PAP, (2) PLP, (3) RLP, and (4) each RLP subtype, including somatic, neuropathic pain, CRPS-like, neuroma, and mosaic neuralgia. The follow-up period of the studies varied; thus, the 6-month time point postamputation was used in the prevalence calculations. Baseline demographic data were collected, including age, sex, and the presence of presurgical limb pain.

### 2.5. Risk of bias assessment

Because the outcome of interest was the prevalence of pain in a single cohort, the risk of bias was assessed using a modified tool specifically designed for assessing bias in uncontrolled studies.^14^ This modified tool consists of 4 questions: (1) do patients represent the whole experience of the investigator or center, (2) was the exposure adequately ascertained, (3) was the outcome adequately ascertained, and (4) is the case described with sufficient details. The risk of bias was reported for each of 4 questions relating to selection, ascertainment, and reporting for each study.

### 2.6. Evidence synthesis

The prevalence of PAP was extracted from each study and meta-analyzed. Statistical analysis was performed after the Freeman–Tukey double arcsine transformation. Results were pooled with random-effects models using the DerSimonian and Laird method and were reported with 95% confidence intervals (CIs). Statistical analyses were performed using R 3.5.0 (R Core Team, 2018),^1,23^ and *P* values <0.05 were considered significant.

## 3. Results

### 3.1. Characteristics of included studies

**Figure 1.**
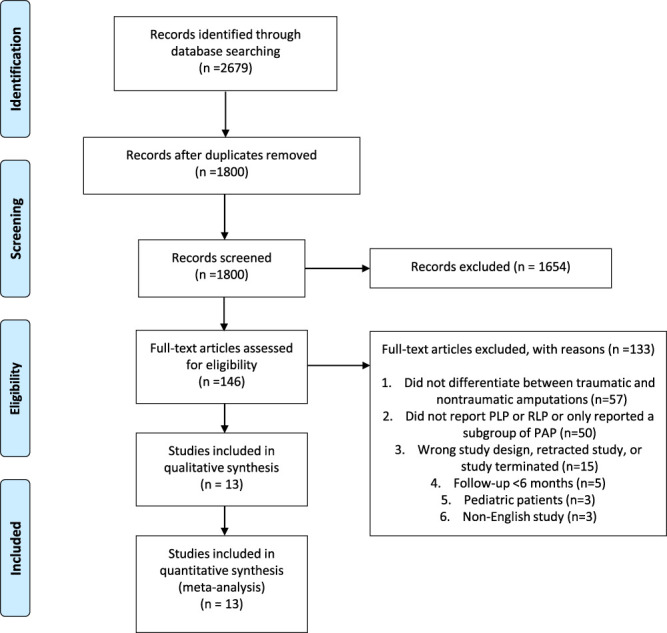
PRISMA flow diagram. PRISMA, Preferred Reporting Items for Systematic Reviews and Meta-Analyses.

A flow diagram of the study selection process is depicted in Figure 1. A total of 13 studies met inclusion criteria (Table 1) The prevalence of all PAP subtypes was reported in 2 studies (n = 534),^13,21^ PLP was reported in 13 studies (n = 1063),^5,7–11,13,15–19,21^ and RLP was reported in 8 studies (n = 783).^7,9,10,13,17–19,21^ One study reported the use of a validated tool, the “Phantom and Residual Limb Phenomena Interview,”^10^ and one study referenced the use of a standardized questionnaire.^21^ No studies reported the prevalence of RLP subtypes.

Study designs included 7 randomized controlled trials,^5,7–9,15,16,18^ 3 prospective cohort studies,^10,17,19^ 2 cross-sectional studies,^13,21^ and one retrospective cohort study.^11^ Included studies were published in Denmark,^15–17^ England,^7,19^ Germany,^10^ Greece,^8^ Norway,^5^ Singapore,^21^ and the United States.^9,11,13,18^ Amputation level was reported for 11 studies.^5,7–9,11,13,15–19^ Seven studies included above-the-knee (AKA) and below-the-knee (BKA) amputations,^7–9,13,16,18,19^ 2 studies included AKA, BKA, and through-the-knee amputations,^15,17^ 1 study included BKA and through-the-knee amputations,^5^ 1 study included AKA,^11^ and 2 studies did not specify the amputation level.^10,21^ All nontraumatic indications for amputation were included.

### 3.2. Risk of bias evaluation

The risk of bias assessment is contained in Appendix (available at http://links.lww.com/PR9/A105)B. The most common sources of bias were related to patient selection (question 1) and adequacy of ascertaining outcomes (question 3).

### 3.3. Prevalence of postamputation pain

**Figure 2.**
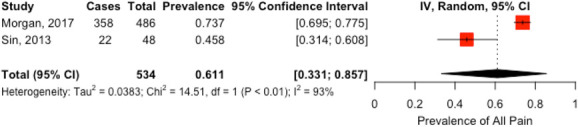
Prevalence of postamputation pain.

Two studies (n = 534)^13,21^ reported the prevalence of all PAP subtypes; the prevalence ranged from 46% to 74% (Fig. 2). The pooled prevalence of PAP was 61% (95% CI, 33%–86%) with high heterogeneity (I^2^ = 93%).

### 3.4. Prevalence of postamputation pain subtypes

#### 3.4.1. Prevalence of phantom limb pain

**Figure 3.**
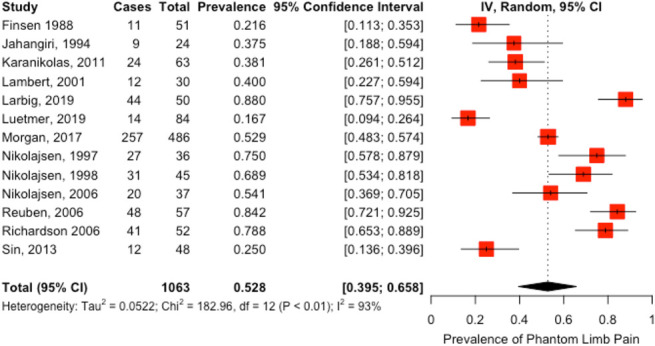
Prevalence of phantom limb pain.

Thirteen studies (n = 1063)^5,7–11,13,15–19,21^ reported the prevalence of PLP; the prevalence ranged from 17% to 88% (Fig. 3). The pooled prevalence of PLP in these studies was 53% (95% CI, 40%–66%) with high heterogeneity (I^2^ = 93%).

Subgroup analysis of PLP prevalence by study design revealed that prospective cohort studies were a statistically significant moderator of heterogeneity (*P* = 0.02) but statistically significant residual heterogeneity remained (*P* < 0.0001). This suggests that study design did not fully account for heterogeneity. Individual subgroup analysis of PLP showed that prevalence by year of publication, country, and country development status were not significant moderators of heterogeneity. Meta-regression with study design, country development status, and year of publication as covariates resulted in significant residual heterogeneity (data not shown).

#### 3.4.2. Prevalence of residual limb

**Figure 4.**
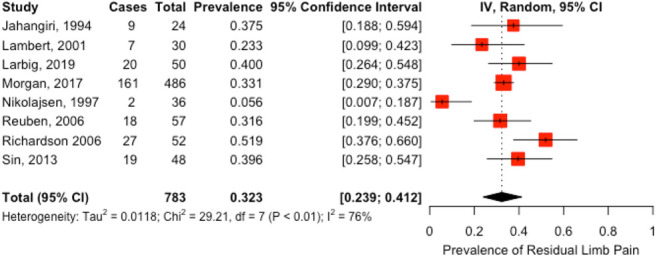
Prevalence of residual limb pain.

Eight studies (n = 783)^7,9,10,13,17–19,21^ reported the prevalence of RLP; the prevalence ranged from 6% to 52% (Fig. 4). The pooled prevalence of RLP was 32% (95% CI 24%–41%) with high heterogeneity (I^2^ = 76%). Meta-regression analyses demonstrated that study design was not a significant moderator of heterogeneity (data not shown).

## 4. Discussion

The primary findings of this systematic review and meta-analysis are (1) the pooled prevalence of PAP is 61%, (2) the pooled prevalence of PLP is 53%, (3) the pooled prevalence of RLP is 32%, and (4) study design is a statistically significant moderator of heterogeneity in studies reporting the prevalence of PLP. The pooled prevalence values were associated with high levels of heterogeneity that warrant further consideration.

The prevalence range of PAP was 28%, but the prevalence range of PLP and RLP were 71% and 46%, respectively. A subgroup analysis of PLP demonstrated that study design was a significant moderator of heterogeneity. Alternatively, subgroup and meta-regression analyses of PLP demonstrated that year of publication, country, and country development status were not significant moderators of heterogeneity. For RLP, study design was not a significant moderator of heterogeneity. Although the multifactorial pathophysiological mechanisms responsible for PAP could contribute to heterogeneity, time since amputation and individual clinical factors could be important contributors. In a longitudinal cross-sectional study from the Netherlands, the prevalence of PLP in patients with lower-extremity amputations 6 months after surgery was 32%.^3^ However, the prevalence declined to 27% at 3.5 years follow-up.^3^ These findings can be contrasted against a cross-sectional study from the United States where the prevalence of PLP in patients who had at least one amputation ranged from 78% to 85% during a mean follow-up period of 26 years.^20^ Approximately 50% of patients reported some improvements in pain, and the remaining 50% reported stable or worsening pain during the follow-up period.^20^ These studies suggest that PAP is a dynamic disease process and the prevalence may vary over time. This may be particularly relevant to patients with RLP due, in part, to the varied and time-dependent pathophysiological mechanisms responsible for the clinical manifestation of symptoms in this important subgroup of patients.

**Table 1:** Study characteristics.

Author	Study design	PAP	PAP subtype		Total patients	Country	Pain duration	Follow-up period	Summary risk of bias
			PLP	RLP					
Finsen,^5^ 1988	RCT	—	11	—	51	Norway	52 (wk)	1 y	Low
Jahangiri,^7^ 1994	RCT	—	9	9	24	England	24	1 y	Moderate
Karanikolas,^8^ 2011	RCT		24	—	63	Greece	24	6 mo	Low
Lambert,^9^ 2001	RCT	—	12	7	30	USA	24	1 y	Low
Larbig,^10^ 2019	Prospective cohort	—	44	20	50	Germany	52	1 y	Low
Luetmer,^11^ 2019	Retrospective cohort	—	14	—	84	USA	—	—	Moderate
Morgan,^13^ 2017	Cross-sectional	358	257	161	486	USA	—	11 y	Low
Nikolajsen,^17^ 1997	Prospective cohort	—	27	2	36	Denmark	42	6 mo	Low
Nikolajsen,^16^ 1998	RCT	—	31	—	45	Denmark	42	6 mo	Moderate
Nikolajsen,^15^ 2006	RCT	—	20	—	37	Denmark	42	6 mo	Low
Reuben,^18^ 2006	RCT	—	48	18	57	USA	—	1 y	Moderate
Richardson,^19^ 2006	Prospective cohort	—	41	27	52	England	24	6 mo	Low
Sin,^21^ 2013	Cross-sectional	22	12	19	48	Singapore	—	—	Low

PAP, postamputation pain; PLP, phantom limb pain; RLP, residual limb pain; RCT, randomized controlled trial.

Individual patient factors may influence of the prevalence of PAP. A cross-sectional study of 122 double amputees revealed high intraindividual concordance for the development of PLP and RLP.^22^ Preoperative pain, sex, and age did not explain concordance in PLP or RLP but the authors reported that recent amputation and short residual limb length were associated with a higher probability of PLP. However, the scope of our systematic review precluded investigating individual factors potentially associated with the development of PAP.

None of the studies included in this systematic review subdivide RLP into the somatic and neuropathic pain subtypes. However, one study described somatic pain and neuroma as possible causes of RLP but the prevalence was not reported.^13^ This observation highlights the need for studies that characterize RLP subtypes in amputees because this subdivision has important treatment implications.

This study has limitations. First, the scope of this systematic review was limited to studies that reported the prevalence of chronic nontraumatic PAP involving the lower extremities. Studies of patients with traumatic amputations alone were excluded because of the risk that treatment of ongoing trauma-related conditions could adversely influence or obscure the identification of PAP. Thus, the prevalence reported in this article may not be applicable to populations of patients with traumatic PAP or populations of patients with upper-extremity PAP and PAP related to amputations of upper-extremity and lower-extremity digits. Second, only 2 studies reported the prevalence of PAP and no studies reported the prevalence of RLP subtypes. Ongoing research using the DPIG-PAPA taxonomy are needed to further investigate the prevalence of PAP and its subtypes. Third, the included studies were published between 1988 and 2019. Although subgroup and meta-regression analyses did not identify significant associations between year of publication and heterogeneity of pooled prevalence rates, it remains possible that advances in surgical technique, perioperative management, and rehabilitation strategies could have influenced the prevalence of PAP. Finally, most differences in the risk of bias were related to selection bias and, to a lesser extent, adequacy of ascertaining outcomes. Thus, these 2 key methodological shortcomings could have influenced the pooled prevalence rates reported in this systematic review.

In conclusion, this systematic review and meta-analysis demonstrate that the prevalence of PAP is high in patients with nontraumatic lower-extremity amputations, but the pooled prevalence rates were associated with high levels of heterogeneity. Aside from a subgroup analysis that suggested study design is a significant moderator of heterogeneity for PLP, other subgroup and meta-regression analyses did not yield significant sources of heterogeneity. Ongoing research that uses the DPIG-PAPA taxonomy is needed to fully delineate the prevalence of PAP and its subtypes.

## Disclosures

The authors have no conflicts of interest to declare.

## Appendix A. Supplemental digital content

Supplemental digital content associated with this article can be found online at http://links.lww.com/PR9/A105.

## Supplementary Material

SUPPLEMENTARY MATERIAL
