# Supplementary material for: Prevalence of postamputation pain and its subtypes: a meta-analysis with meta-regression
Source: Pain Rep. 2021 May 4;6(1):e918. doi: 10.1097/PR9.0000000000000918 (PMC8108594; doi:10.1097/PR9.0000000000000918)
Supplement: SUPPLEMENTARY MATERIAL [file painreports-6-e918-s001.docx]

**Supplemental Material: Meta-Regressions**

**1. Meta-regression with study design, country development status, and year of publication as covariates for phantom limb pain.**

1.a. Study Design

> metareg(phantommeta, ~ studydesign)

Mixed-Effects Model (k = 13; tau^2 estimator: DL)

tau^2 (estimated amount of residual heterogeneity):     0.0396 (SE = 0.0221)

tau (square root of estimated tau^2 value):             0.1991

I^2 (residual heterogeneity / unaccounted variability): 88.63%

H^2 (unaccounted variability / sampling variability):   8.79

R^2 (amount of heterogeneity accounted for):            24.03%

Test for Residual Heterogeneity:

QE(df = 9) = 79.1314, p-val < .0001

Test of Moderators (coefficients 2:4):

QM(df = 3) = 10.4262, p-val = 0.0153

Model Results:

                estimate      se     zval    pval    ci.lb   ci.ub

intrcpt           0.6796  0.1455   4.6702  <.0001   0.3944  0.9648  ***

studydesignPC     0.4320  0.1903   2.2700  0.0232   0.0590  0.8050    *

studydesignRC    -0.2538  0.2525  -1.0052  0.3148  -0.7488  0.2411

studydesignRCT    0.1032  0.1665   0.6200  0.5353  -0.2231  0.4295

Signif. codes:  0 ‘***’ 0.001 ‘**’ 0.01 ‘*’ 0.05 ‘.’ 0.1 ‘ ’ 1

1.b. Country development status

> metareg(phantommeta, ~ developstat)

Mixed-Effects Model (k = 13; tau^2 estimator: DL)

tau^2 (estimated amount of residual heterogeneity): 0.0515 (SE = 0.0320)

tau (square root of estimated tau^2 value): 0.2268

I^2 (residual heterogeneity / unaccounted variability): 93.44%

H^2 (unaccounted variability / sampling variability): 15.24

R^2 (amount of heterogeneity accounted for): 1.40%

Test for Residual Heterogeneity:

QE(df = 11) = 167.6146, p-val < .0001

Test of Moderators (coefficient 2):

QM(df = 1) = 1.5338, p-val = 0.2155

Model Results:

estimate se zval pval ci.lb ci.ub

intrcpt 0.8361 0.0687 12.1675 <.0001 0.7014 0.9708 ***

developstatunder -0.3067 0.2476 -1.2385 0.2155 -0.7921 0.1787

Signif. codes: 0 ‘***’ 0.001 ‘**’ 0.01 ‘*’ 0.05 ‘.’ 0.1 ‘ ’ 1

1.c. Year of publication

> metareg(phantommeta, ~ yr)

Mixed-Effects Model (k = 13; tau^2 estimator: DL)

tau^2 (estimated amount of residual heterogeneity): 0.0625 (SE = 0.0354)

tau (square root of estimated tau^2 value): 0.2500

I^2 (residual heterogeneity / unaccounted variability): 93.99%

H^2 (unaccounted variability / sampling variability): 16.63

R^2 (amount of heterogeneity accounted for): 0.00%

Test for Residual Heterogeneity:

QE(df = 11) = 182.9327, p-val < .0001

Test of Moderators (coefficient 2):

QM(df = 1) = 0.0523, p-val = 0.8190

Model Results:

estimate se zval pval ci.lb ci.ub

intrcpt -2.6699 15.2216 -0.1754 0.8608 -32.5037 27.1639

yr 0.0017 0.0076 0.2288 0.8190 -0.0131 0.0166

Signif. codes: 0 ‘***’ 0.001 ‘**’ 0.01 ‘*’ 0.05 ‘.’ 0.1 ‘ ’ 1

**2. Similarly, in the residual limb pain results section, the meta-regression has been submitted as supplemental material. The contents of this supplement are duplicated below:**

2.a. Study design

> metareg(residmeta, ~ studydesign)

Mixed-Effects Model (k = 8; tau^2 estimator: DL)

tau^2 (estimated amount of residual heterogeneity): 0.0239 (SE = 0.0193)

tau (square root of estimated tau^2 value): 0.1546

I^2 (residual heterogeneity / unaccounted variability): 82.69%

H^2 (unaccounted variability / sampling variability): 5.78

R^2 (amount of heterogeneity accounted for): 0.00%

Test for Residual Heterogeneity:

QE(df = 5) = 28.8782, p-val < .0001

Test of Moderators (coefficients 2:3):

QM(df = 2) = 0.1565, p-val = 0.9247

Model Results:

estimate se zval pval ci.lb ci.ub

intrcpt 0.6451 0.1152 5.5997 <.0001 0.4193 0.8710 ***

studydesignPC -0.0537 0.1519 -0.3533 0.7238 -0.3514 0.2441

studydesignRCT -0.0534 0.1540 -0.3470 0.7286 -0.3552 0.2484

Signif. codes: 0 ‘***’ 0.001 ‘**’ 0.01 ‘*’ 0.05 ‘.’ 0.1 ‘ ’ 1
